# Supplementary material for: Highly accurate assembly polishing with DeepPolisher
Source: bioRxiv. 2024 Sep 19:2024.09.17.613505. Preprint. [Version 1] doi: 10.1101/2024.09.17.613505 (PMC11429912; doi:10.1101/2024.09.17.613505)
Supplement: Supplement 2 [file NIHPP2024.09.17.613505v1-supplement-2.pdf]

bioRxiv preprint doi: https://doi.org/10.1101/2024.09.17.613505; this version posted September 19, 2024. The copyright holder for this preprint (which was not certified by peer review) is the author/funder, who has granted bioRxiv a license to display the preprint in perpetuity. It is made available under aCC-BY 4.0 International license.

Supplementary figures:

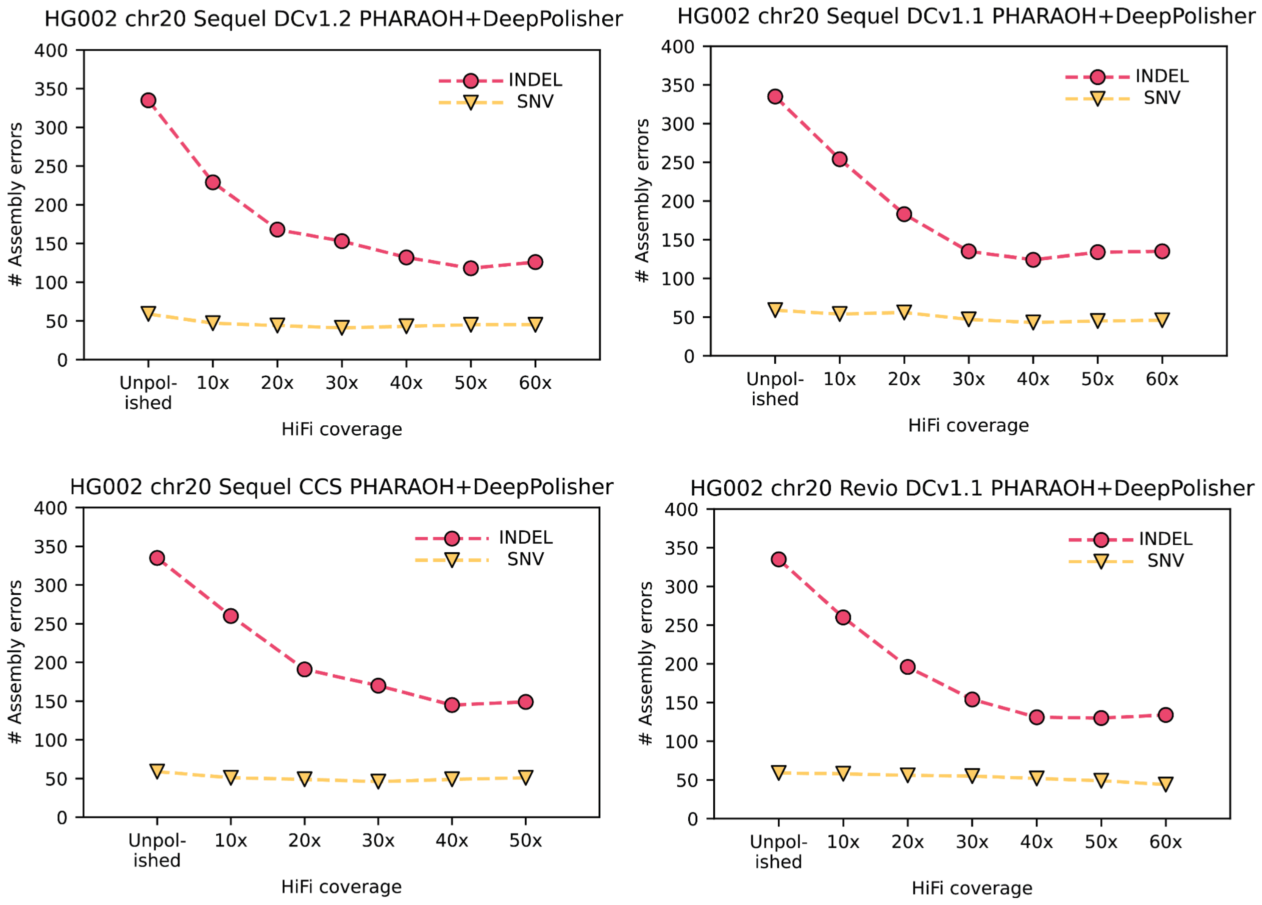

Supplementary Figure 1: HG002 chr20 GIAB variant calling performance for other HiFi read versions, across different coverages. Total GIAB variant calling (assembly) errors for different HiFi read coverages, with indel errors represented in pink circles and SNV errors in yellow triangles

---

bioRxiv preprint doi: https://doi.org/10.1101/2024.09.17.613505; this version posted September 19, 2024. The copyright holder for this preprint (which was not certified by peer review) is the author/funder, who has granted bioRxiv a license to display the preprint in perpetuity. It is made available under aCC-BY 4.0 International license.

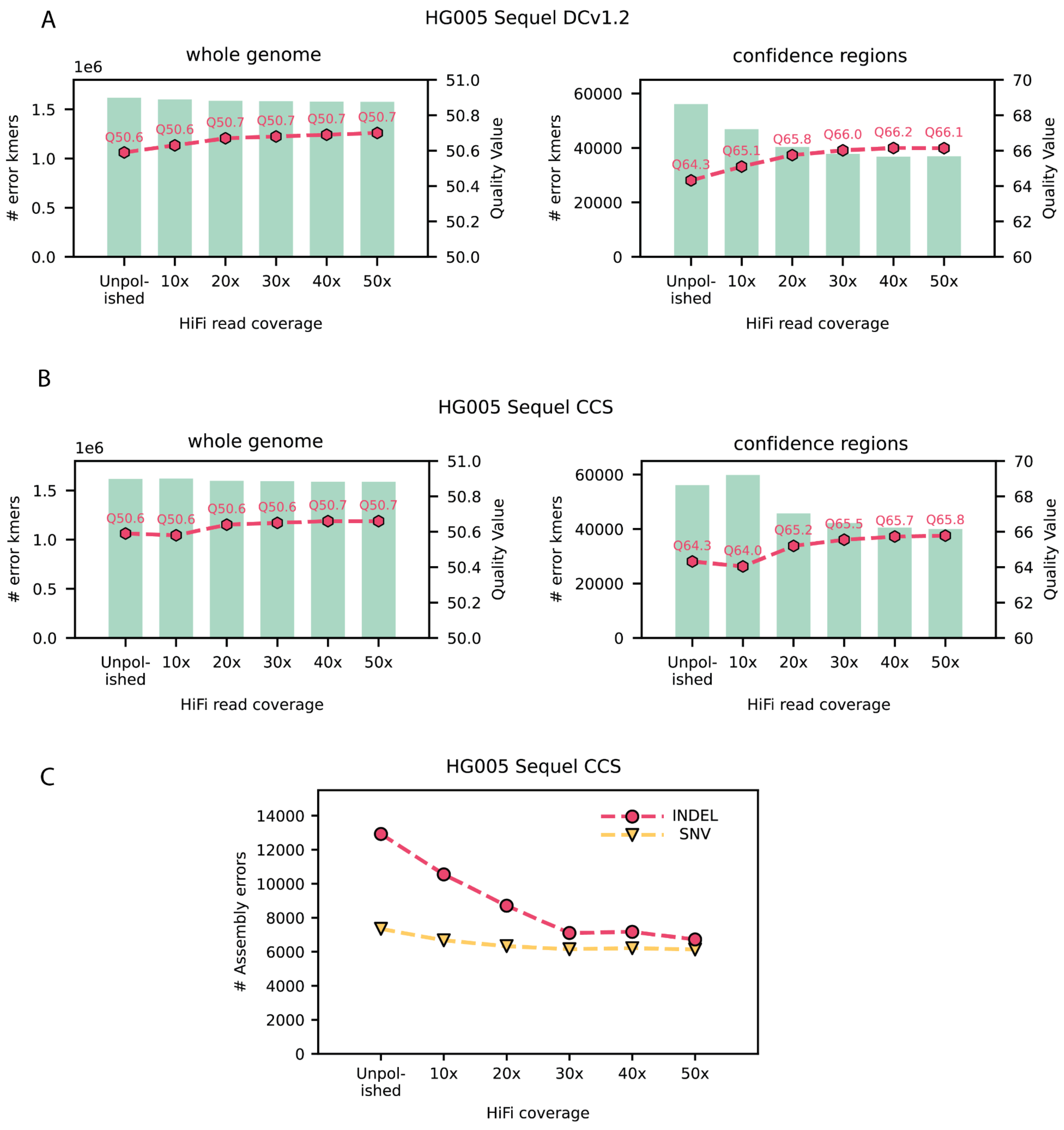

Supplementary Figure 2: HG005 data performance across multiple coverages and read technologies. A) Bar plots represent number of error k-mers (left y axis) for HiFi Sequel DCv1.2 at different read coverages, with the corresponding QV plotted on the right y axis for whole genome (left panel) and for the confidence regions (right panel) **B)** The same for HiFi Sequel CCS data. **C)** Total GIAB variant calling (assembly) errors for different HiFi read coverages, with indel errors represented in pink circles and SNV errors in yellow triangles

---

bioRxiv preprint doi: https://doi.org/10.1101/2024.09.17.613505; this version posted September 19, 2024. The copyright holder for this preprint (which was not certified by peer review) is the author/funder, who has granted bioRxiv a license to display the preprint in perpetuity. It is made available under aCC-BY 4.0 International license.

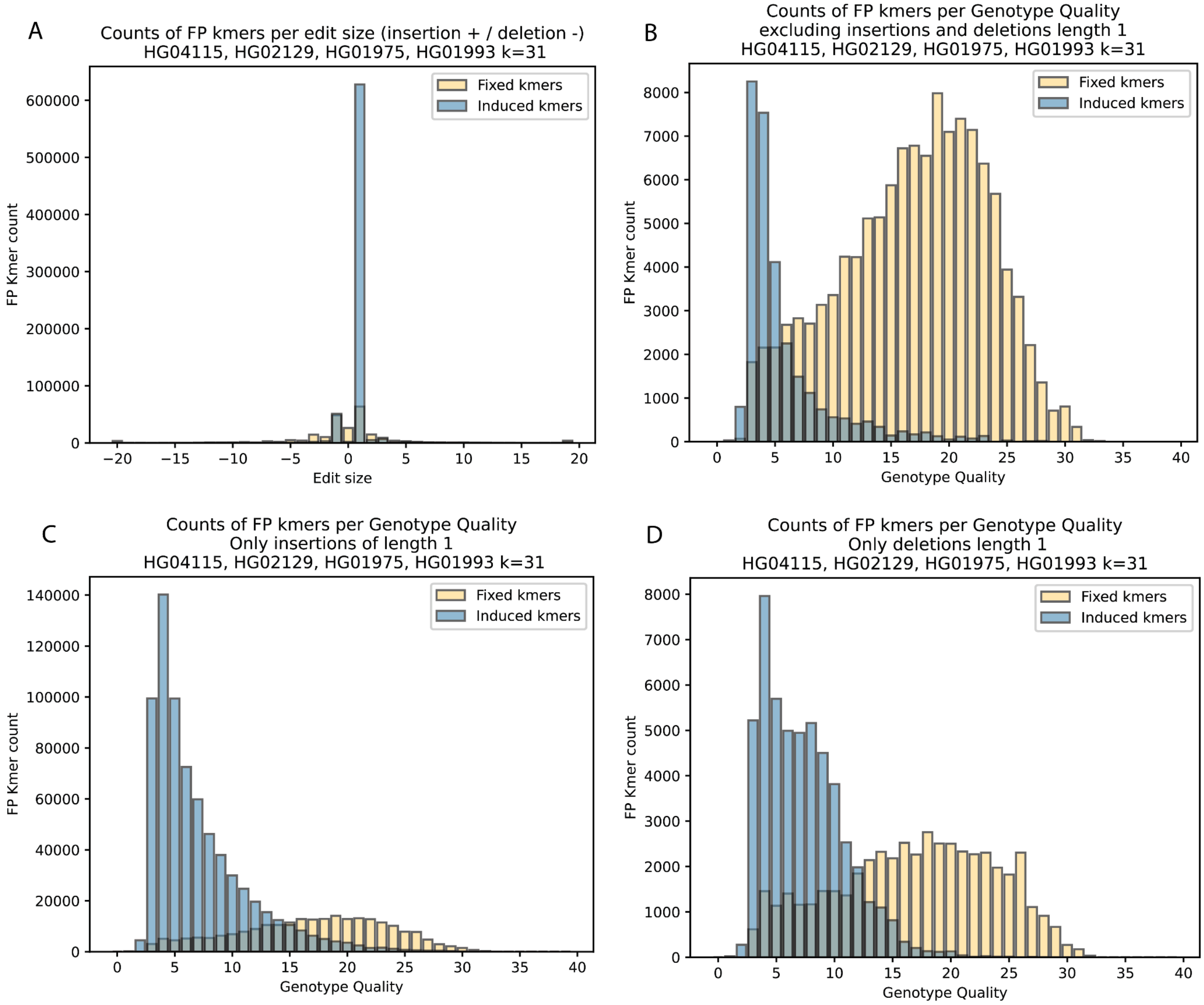

Supplementary Figure 3: Optimizing GQ filters for DeepPolisher

A) Count of error (FP) k-mers per polishing edit size, with error k-mers fixed by polishing edit in yellow and error k-mers induced by polishing edit in blue. **B)** Counts of error (FP) k-mers per genotype quality of polishing edit, excluding insertions and deletions of length 1, and for **C**) the same for only insertions of length 1, and **D)** for deletions of length 1.

bioRxiv preprint doi: https://doi.org/10.1101/2024.09.17.613505; this version posted September 19, 2024. The copyright holder for this preprint (which was not certified by peer review) is the author/funder, who has granted bioRxiv a license to display the preprint in perpetuity. It is made available under aCC-BY 4.0 International license.

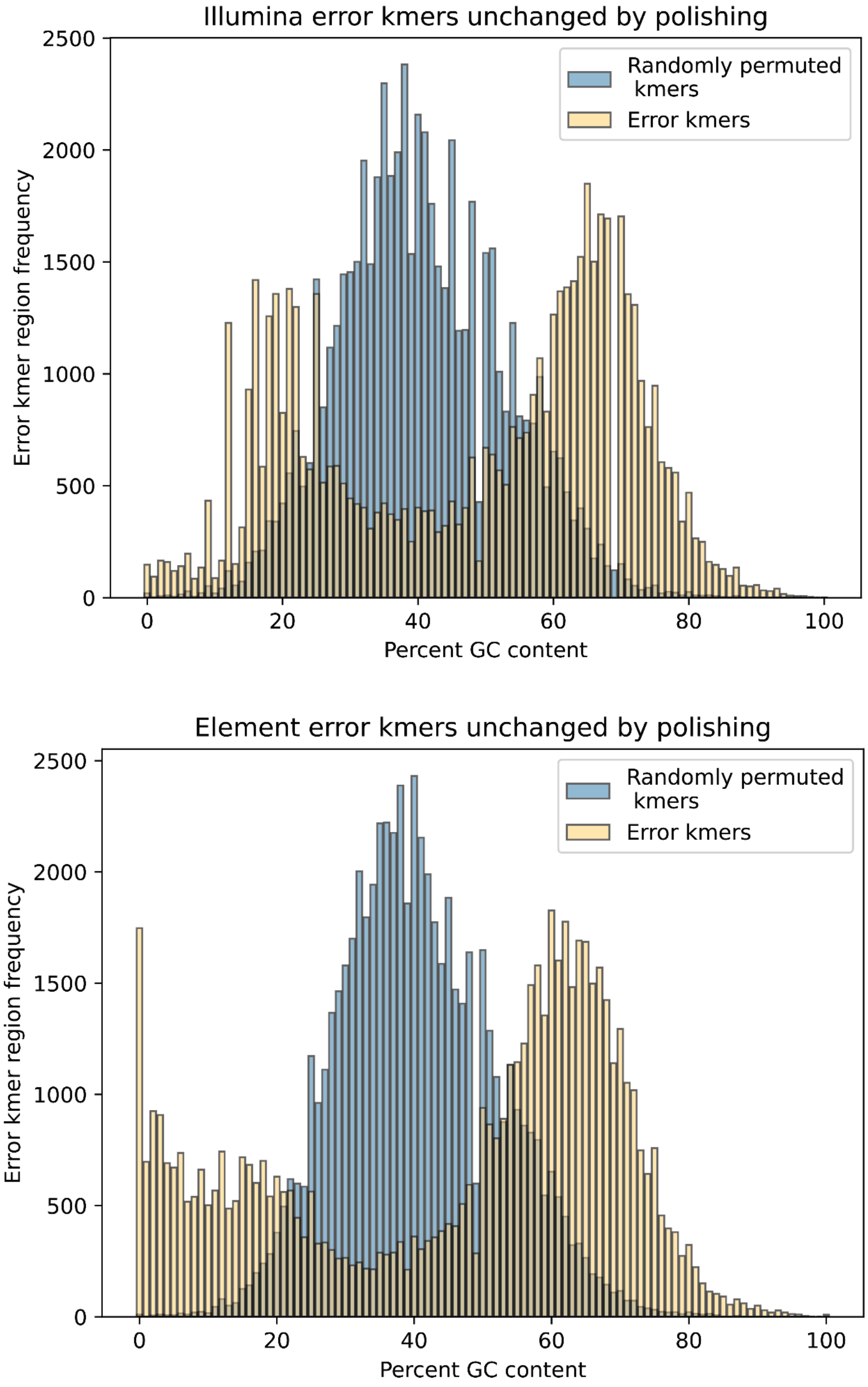

Supplementary Figure 4: Percent GC content of error k-mers unchanged by polishing and randomly permuted k-mers. Frequency of merged error k-mer regions per varying GC contents. In yellow shows the actual observed error k-mers produced by Merqury, in blue are randomly permuted k-mers of the same size across the genome. Top panel is for Illumina, bottom panel for Element cloudbreak.
